# Dinoflagellate nucleus contains an extensive endomembrane network, the nuclear net

**DOI:** 10.1038/s41598-018-37065-w

**Published:** 2019-01-29

**Authors:** Gregory S. Gavelis, Maria Herranz, Kevin C. Wakeman, Christina Ripken, Satoshi Mitarai, Gillian H. Gile, Patrick J. Keeling, Brian S. Leander

**Affiliations:** 1 School of Life Sciences, Arizona State University, Tempe, AZ 85287-4501 USA; 2 Department of Botany, University of British Columbia, Vancouver, BC V6T-1Z4 Canada; 3 Department of Zoology, University of British Columbia, Vancouver, BC V6T-1Z4 Canada; 4 Department of Biological Sciences: Faculty of Science, Hokkaido University, Sapporo, 060-0810 Japan; 5 Okinawa Institute of Science and Technology, Graduate University, Kunigami-gun, Okinawa, 904-0495 Japan; 6 Marine Biophysics Unit, Okinawa Institute of Science and Technology, Kunigami-gun, Okinawa, 904-0495 Japan

## Abstract

Dinoflagellates are some of the most common eukaryotic cells in the ocean, but have very unusual nuclei. Many exhibit a form of closed mitosis (dinomitosis) wherein the nuclear envelope (NE) invaginates to form one or more trans-nuclear tunnels. Rather than contact spindles directly, the chromatids then bind to membrane-based kinetochores on the NE. To better understand these unique mitotic features, we reconstructed the nuclear architecture of *Polykrikos kofoidii* in 3D using focused ion beam scanning electron microscopy (FIB-SEM) in conjunction with high-pressure freezing, freeze-substitution, TEM, and confocal microscopy. We found that *P. kofoidii* possessed six nuclear tunnels, which were continuous with a reticulating network of membranes that has thus far gone unnoticed. These membranous extensions interconnect the six tunnels while ramifying throughout the nucleus to form a “nuclear net.” To our knowledge, the nuclear net is the most elaborate endomembrane structure described within a nucleus. Our findings demonstrate the utility of tomographic approaches for detecting 3D membrane networks and show that nuclear complexity has been underestimated in *Polykrikos kofoidii* and, potentially, in other dinoflagellates.

## Introduction

Dinoflagellate nuclei (dinokarya) have long fascinated cell biologists because of their bizarre features. They contain some of the largest eukaryotic genomes, housed in dozens to hundreds of chromosomes that remain permanently condensed throughout the cell cycle^1,2^. The chromosomes are characteristically dense, some existing in a “liquid crystalline state,” while all seem to lack nucleosomes^3–5^. Phylogenomic reconstructions,^6,7^ and recent experimental work^8^, suggest that nucleosomes were lost in the common ancestor of all dinoflagellates and that their DNA packing role was taken over by nucleoproteins acquired from a virus. Dinoflagellate genome architecture is highly unusual, with genes arranged unidirectionally, often as tandem repeats, and the vast majority of genomic DNA is noncoding^9–11^. The sparse coding regions probably occupy “loops” of DNA at the chromosome periphery, which are organized by histone-like proteins of bacterial origin^12–14^. In the past decade, new approaches have illuminated the unusual arrangement of proteins and DNA within dinoflagellate chromosomes, as well as their coordination throughout the cell cycle^15–17^. However, much less attention has been paid to the membranes that surround them (i.e., the nuclear envelope).

The nuclear envelope (NE) and the endoplasmic reticulum—which are continuous—together constitute the most conserved organelle(s) in eukaryotic history, given that even mitochondria, the Golgi apparatus, and flagella have been abandoned in certain eukaryotic lineages^18–20^. Besides acting as a gatekeeper to the nucleus, the dinoflagellate NE takes on an unusual conformation during mitosis, called “dinomitosis” in core dinoflagellates (i.e., dinoflagellates other than *Oxyrrhis* and syndinians). By definition, dinomitosis is a form of closed mitosis, since the NE never breaks down. Instead, it pinches inward at each nuclear pole to form a “tunnel” through the nucleus; essentially turning the nucleus into a toroidal shape resembling a doughnut. By traversing the tunnel, cytoplasmic spindles are able to cross the dinokaryon without ever entering the nucleoplasm. This stands in contrast to most organisms with closed mitosis, which use either intra-nuclear or NE-spanning spindles to separate the chromatids^21–23^. Uniquely, dinomitotic chromatids never directly contact the spindles; instead they attach to membrane-bound kinetochores on the inner NE membrane. The chromatids then migrate to opposite ends of the membranous tunnel^24^. Once segregation is complete, the nucleus divides and the tunnel pinches apart in the middle, returning each daughter nucleus to a spherical shape^25–27^. Where studied, early-branching dinomitotic lineages have a single tunnel (e.g., *Noctiluca scintillans, Syndinium* sp., and *Haplozoon*—if it is early branching), while core dinoflagellates diverging after *N. scintillans* (e.g. *Amphidinium carterae, Prorocentrum minium, Heterocapsa sp*., and *Crypthecodinium cohnii*) have multiple tunnels in parallel—with a maximum of five, as described in *C. cohnii*^28^. Thus, like the evolution of dinoflagellate chromosomes, mitosis has become increasingly unusual and elaborate in some dinoflagellates^29^. The details of dinomitosis deserve deeper investigation, given that dinoflagellate cell cycles proceed at a curiously slow pace compared to other algae^30^, which has important ecological consequences in the marine plankton, where dinoflagellates are among the most common algae and consumers^31,32^.

**Figure 1:**
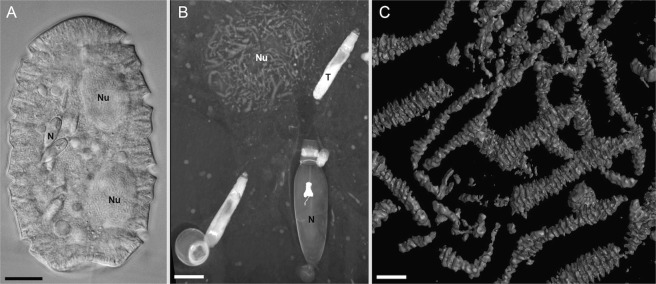
Cellular features of *Polykrikos kofoidii*. (**A**) Differential interference contrast (DIC) light micrograph of a *P. kofoidii* pseudocolony, which is defined by the presence of two nuclei (Nu) and nematocysts (N). (**B**) Maximum intensity projection of several FIB-SEM sections showing a nematocyst (N), a taeniocyst (T) and the side of a nucleus (Nu). (**C**) FIB-SEM surface reconstruction of several chromosomes. Scale bar A = 30 µm, B = 10 µm, C = 2 µm.

Here, we investigated the complexity of nuclear membranes in *Polykrikos kofoidii*, a large, predatory dinoflagellate. While most dinomitotic studies have focused on cells with modestly sized nuclei^33–36^ (e.g., *Crypthecodinium cohnii*, in which nuclei are ~10 µm wide and contain 99 to 110 chromosomes), *P. kofoidii* is an interesting subject because its nuclei are giant—at ~40 µm in diameter—and each contains hundreds of chromosomes. Moreover, *P. kofoidii* is “pseudocolonial” with eight flagella and two nuclei per cell (Fig. 1A), compared to the typical complement of two flagella and one nucleus per dinoflagellate cell^37^. Polykrikoids are ecologically important as voracious predators of harmful algal blooms, which they capture using elaborate secretory organelles (Fig. 1B)^38–40^, and can consume multiple cells of chain-forming prey at a time, in part facilitated by the large size of their pseudocolonies^41,42^. The nuclei in *P. kofoidii* are correspondingly giant, and each is tethered to the nearest pair of flagellar basal bodies by fibrous ribbons. Previous studies have shown its NE to possess bubble-like convexities (“nuclear chambers”) and multiple tunnels during mitosis^43^.

We investigated dinomitotic membrane architecture in greater depth, by using focused ion beam scanning electron microscopy (FIB-SEM) to digitally reconstruct a giant nucleus from *P. kofoidii* in 3D, and confirmed our findings using immuno-fluorescence confocal microscopy and TEM on a dozen additional specimens. Prior to this study, only one dinoflagellate nucleus had been modeled in 3D; a mitotic dinokaryon in *C. cohnii*, which was inferred from serial section transmission electron microscopy (TEM) on a single specimen prepared using standard chemical fixation^28^. Our study is the first attempt to reconstruct a dinokaryon from FIB-SEM data, which we used in combination with improved fixation techniques—high-pressure freezing and freeze-substitution—specifically chosen to minimize membrane artifacts. In addition to confirming known features of dinoflagellate nuclei (e.g., NE tunnels), we also uncovered a novel membranous network, which ramified throughout the nucleus into a sprawling “nuclear net” that interlinked the six tunnels. This web-like, membranous structure represents a new level of complexity for the dinokaryon.

## Results

**Figure 2:**
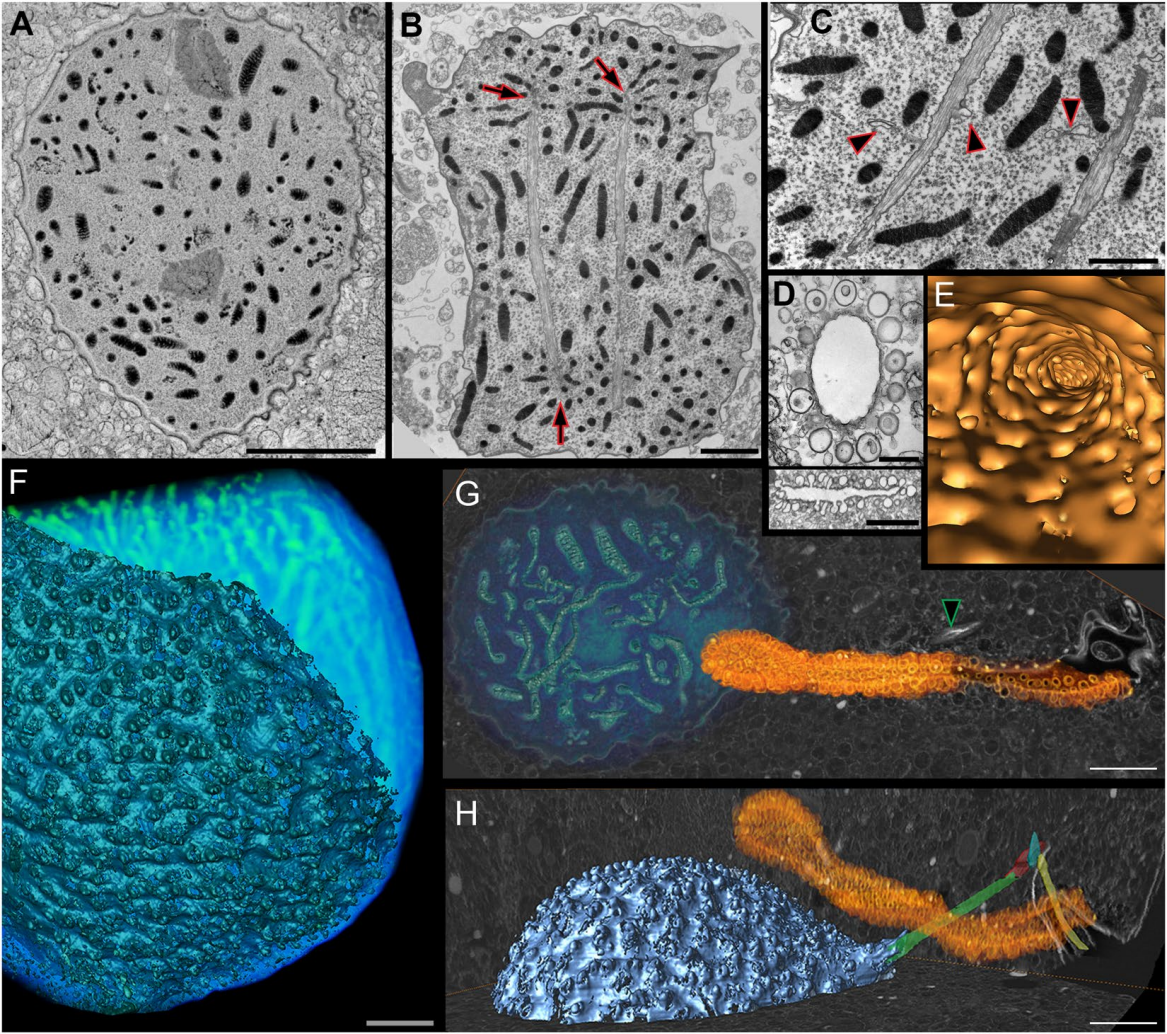
Nucleus and associated membranes. (**A**) FIB-SEM section of a *Polykrikos kofoidii* nucleus fixed by freeze substitution (image has been inverted). (**B**) TEM section of a chemically fixed nucleus of *P. kofoidii*, with chromosomes congregating around dinomitotic tunnels (arrows). (**C**) Longitudinal TEM section of the dinomitotic tunnels and strands of the nuclear net (arrowheads). (**D**, top) Transverse TEM section of the pusule, showing the diverticula lying just under the lumen of the collecting duct. (**D**, bottom): Longitudinal TEM section of the pusule, showing some diverticula meeting the collecting duct. (**E**) FIB-SEM surface rendering of the pusule, which is viewed from the inside, facing towards the proximal end of the lumen. (**F)** FIB-SEM surface rendering of the nuclear envelope (dark blue), overlying a volume rendering of the chromosomes (bright blue). The bumpy texture of the nuclear envelope reflects the nuclear chambers. **(G,H**) FIB-SEM section (gray scale) overlaid with a volume rendering of the pusule (orange) and nucleus (blue). The nucleus is tethered to the flagellum by a fibrous ribbon (green arrowhead in G, green fiber in H). The longitudinal flagellum (yellow) is accompanied by two striated rootlets (red and blue fibers). Scale bar A = 10 µm, B = 4 µm, C & D = 2 µm, F-H = 5 µm.

**Figure 3:**
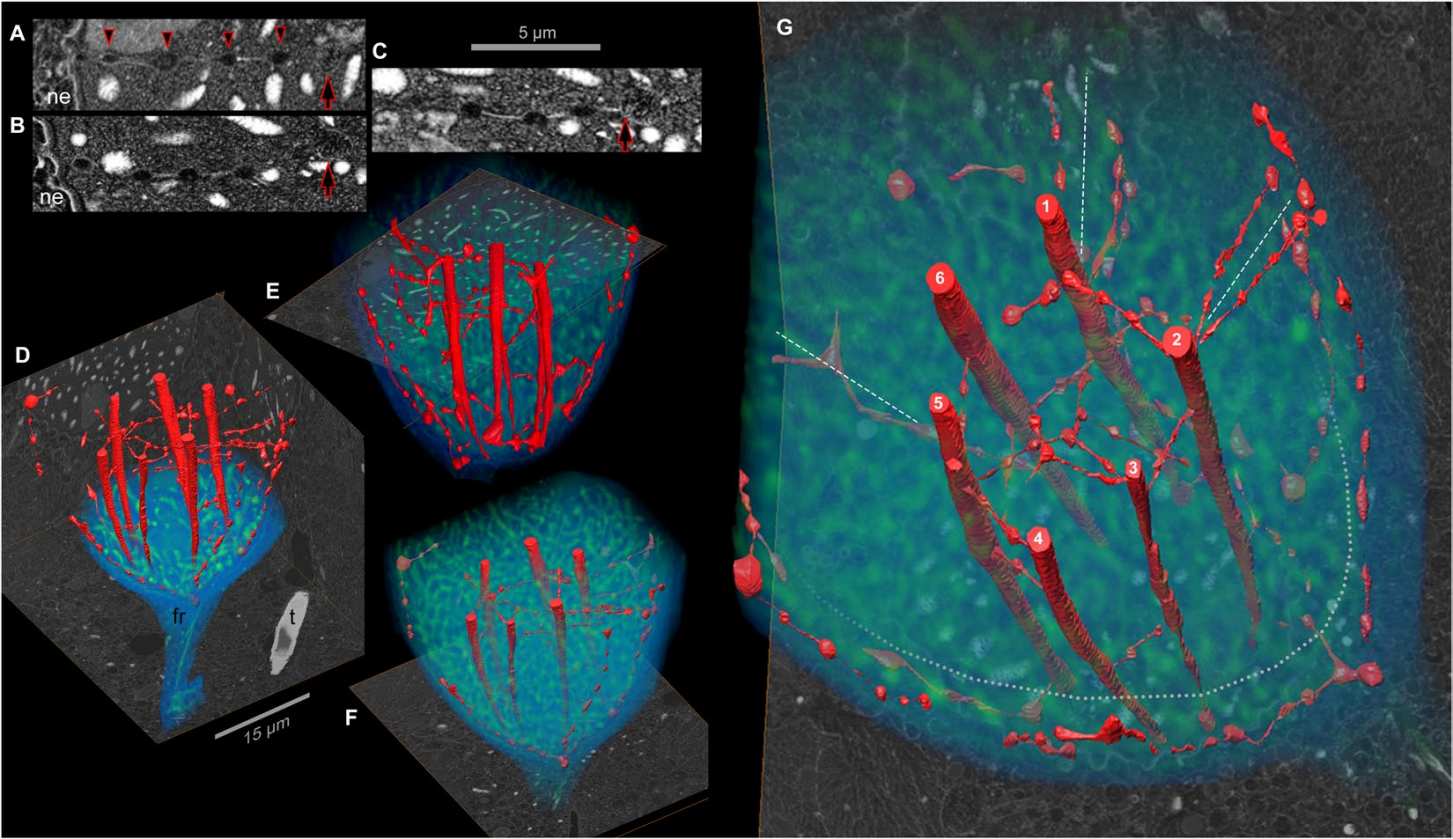
The nuclear net. (**A**–**C**) FIB-SEM sections of the nucleus revealing strands of the nuclear net, which has regularly-spaced membranous nodes (arrowheads). These strands reach from the nuclear envelope (ne) to a dinomitotic tunnel (arrow), which is seen in transverse section. Chromosomes are visible as white fingerprint-like spots. (**D**–**F**) FIB-SEM sections (gray scale) overlain by surface renderings of the nuclear net (red) and volume renderings (blue) of the region surrounding the nucleus and fibrous ribbon (fr). A taeniocyst (t) is also shown. (**G**) Six dinomitotic tunnels (numbered 1–6) mark the core of the nuclear net. Dashed white lines indicate regions where the nuclear net appears to divide the nucleus into quadrants. Membranes positioned outside of the dotted gray line are part of the peripheral nuclear net that lies beneath the surface of the nuclear envelope.

We first conducted a TEM investigation on several chemically-fixed mitotic cells of *Polykrikos kofoidii* and confirmed previously noted^43^ features, such as (1) chromosomes that associate with the NE of dinomitotic tunnels (Fig. 2B,C), as well as (2) elaborate infoldings of the plasma membranes that are each called a “pusule” (Figs 2D,E and 3) the presence of “fibrous ribbons” that tether each nucleus to the nearest pair of flagellar basal bodies (Fig. 2G,H). We also confirmed that the nucleus is studded with bubble-like convexities of the NE known as “nuclear chambers” (Fig. 2F). However, we noticed a previously overlooked feature; thin, membranous interconnections between the dinomitotic tunnels (Fig. 2C, arrowheads). In order to verify that these were not artifacts of chemical fixation, we then preserved a pseudocolony using high-pressure freezing followed by freeze substitution—improved fixation methods that minimize extraction and membrane distortion. We subsequently imaged this pseudocolony using FIB-SEM.

While the membranous interconnections prepared by chemical fixation did not appear highly organized (Fig. 2C), FIB-SEM in conjunction with high-pressure freezing revealed that these gossamer interconnections had a regular arrangement, with periodic swellings that resembled dew-drops on a spider web (Figs 2A and 3A–C). Tomographic FIB-SEM sections were then overlaid and digitally reconstructed in 3D, allowing for these membranous connections to be better visualized (Fig. 3D–G). This revealed a sprawling network of membranous strands that ran between all six dinomitotic tunnels, as well as between the tunnels and the walls of the nucleus—ramifying into a membranous web, which we called the “nuclear net.” The inner portion of the nuclear net (strands between the six dinomitotic tunnels) was relatively well organized—with evenly spaced membranous swellings (or “nodes”) along the strands—while strands in the outer region were thicker and less orderly. Each strand was lined by two membranes (Supplementary Figure 1), which were continuous with those lining the dinomitotic tunnels. Since the dinomitotic membranes are infoldings of the NE^43^, the nuclear net is itself part of the NE, by extension. The lumen of the nuclear net and the dinomitotic tunnels is also continuous (Supplementary Figure 1). Thus, it would seem that cytoplasm could flow from the dinomitotic tunnels into parts of the nuclear net, though access to the innermost strands of the net may be occluded by the membranous constrictions between the nodes (Fig. 3B,C). The orientation of strands appeared somewhat random, though many fused with dinomitotic tunnels at approximately right angles, and strands were most prevalent near the future division plane of the nucleus.

**Figure 4:**
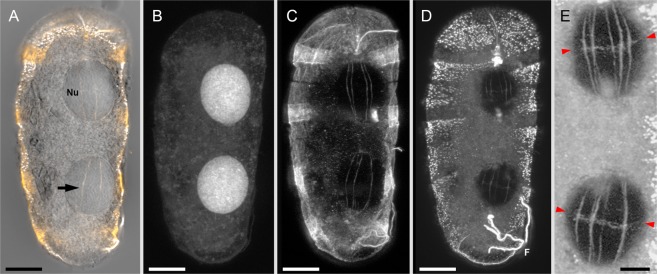
Immunofluorescence of the nucleoskeleton in *P. kofoidii*. (**A**) DIC image overlaid by stacked confocal optical sections of fluorescently stained tubulin (orange) and centrin (white) to show the cytoplasmic tunnels (arrow) that pass through each nucleus (Nu). (**B–E**) Sub-stacks of the same cell with labeled DNA (**B**), tubulin (**C**), and centrin (**D,E**), seen as a maximum intensity projection. (**E**) Image is contrast-enhanced to show the lateral extensions of the nuclear net (between red arrows). Scale bar in A-D = 30 µm, and E = 10 µm.

**Figure 5:**
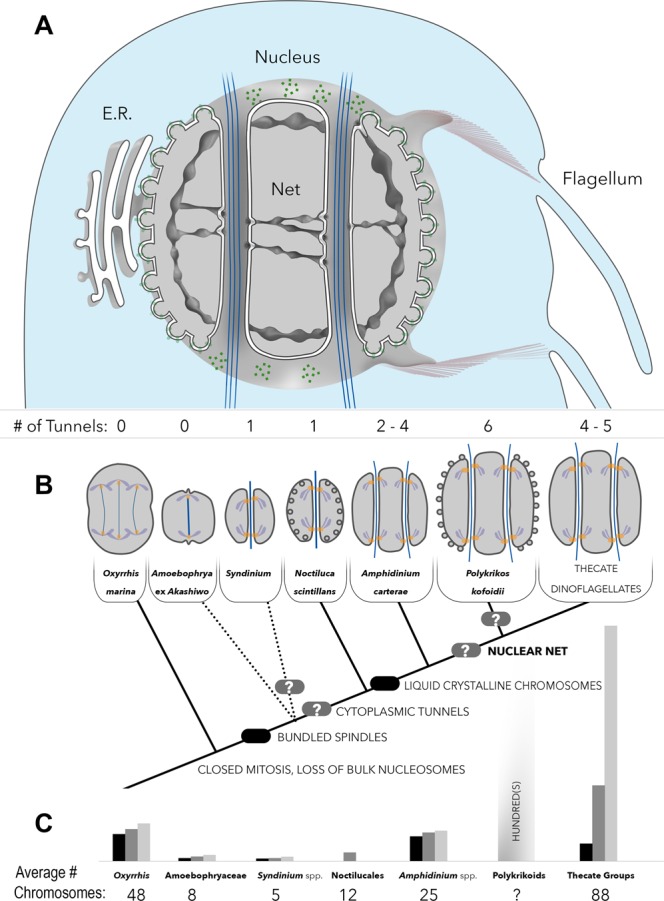
Culmination of nuclear complexity in dinoflagellates. (**A**) A diagrammatic model of the nuclear net. White = endomembrane space; light gray = nucleoplasm; light blue = cytoplasm; dark blue = spindles; green = nuclear pores; purple = fibrous ribbons. (**B**) Different forms of mitosis mapped onto the phylogeny of dinoflagellates (based on Janouškovec *et al*.)^7^, Nuclei are diagrammed in cross section, and shown at late telophase (the nuclear net is omitted for clarity). Peripheral circles represent the nuclear chambers, which are concave in *Noctiluca* and convex in *Polykrikos*. Light gray = nucleoplasm, dark gray = nuclear envelope, purple = chromosomes, blue = dark spindles, orange = kinetochores. Other forms of mitotic nuclei are not illustrated here if they either lack sufficient information to be diagrammed or were observed in groups of uncertain phylogenetic position. Rounded rectangles denote the position of ancestral character traits. (**C**) The average chromosome count for each dinoflagellate group (based on Loeblich 1976)^33^. The bar graph depicts the minimum (black), mean (dark grey), and maximum (light grey) chromosome counts.

We sought to confirm the presence of the nuclear net using light microscopy and also to investigate whether it contains microtubules or other proteins contributing to nucleoskeletal integrity. To this end, we imaged multiple cells of *P. kofoidii* through confocal laser microscopy. Fluorescent immunolabelling was used to localize molecules of interest; DNA (Fig. 4B), tubulin (Fig. 4C), and centrin (Fig. 4D,E). As expected, the tubulin antibody labeled multiple spindle microtubules traversing the nucleus (Fig. 4A,C), ostensibly via the dinomitotic tunnels. The centrin antibody labeled not only these spindles, but also fibrous structures running perpendicularly between them (Fig. 4D,E). This corresponds to our observations of the nuclear net through FIB-SEM, given that the strands of the nuclear net were most concentrated in this region—the future division plane of the nucleus (Fig. 5A). The fact that our antibodies also labeled expected targets (the nucleus, cell cortex, and transverse flagellum for DNA, tubulin, and centrin, respectively) supports the accuracy of our staining protocol.

## Discussion

In sum, our investigations using TEM, FIB-SEM, and confocal microscopy across a dozen specimens together support the existence of an elaborate endomembrane network within the nucleus of *P. kofoidii*. The use of different imaging modalities, fixation methods, and cells collected from several locations over two years suggests that this intricate structure is not an artifact. Critically, comparison of polykrikoid nuclear net ultrastructure fixed through both high-pressure freezing (Supplementary Figure 2A,B) and chemical fixation (Supplementary Figure 2C,F) showed that it is partially disrupted when prepared through standard chemical fixation. Thus, it is likely that the nuclear net was overlooked in the two previous TEM studies^43,44^ of nuclei in *P. kofoidii* due to the deformation of its fragile membranous strands during chemical fixation. Revisiting a study by Spector & Treimer (1981) on *P. kofoidii* that were collected from the same site as our specimens, we again found evidence of disrupted nuclear nets; i.e., double-membrane-lined strands protruding from the dinomitotic tunnels (tracings in Supplementary Fig. 1E,F)^44^. Compared to our freeze-substituted preparations, these strands were sparse and poorly organized, but were similar to those we had initially prepared through standard chemical fixation (Supplementary Fig. 2C,D). It is not surprising that the nuclear net is only intact when prepared by high-pressure freezing and freeze substitution, as cryofixation immobilizes cell ultrastructure almost instantaneously, and thereby avoids the membrane distortion that is common in standard, chemically-fixed specimens^45–47^.

To see if nuclear nets could be an overlooked feature in dinoflagellates beyond *P. kofoidii*, we surveyed other published TEM studies of the dinokaryon. We noticed clear double-membrane-lined strands extending from the dinomitotic tunnels in *Prorocentrum minimum*^48^, *Crypthecodinium cohnii*^24,28^*, Heterocapsa* sp.^49^ and *Kryptoperidinium foliaceum*^50^. Interestingly, strands were only observed in studies of mitotic nuclei and—as in our studies and those of Spector and Treimer (1981)—they were always present as outgrowths of the dinomitotic tunnels. Several of these strands interlinked adjacent dinomitotic tunnels^24,28,48^ (Supplementary Figure 2G,H). Perhaps consequently, we did not observe them in the published images of mitotic nuclei with only one tunnel, such as *Noctiluca*^51^, *Oodinium*^52^, *Haplozoon*^53^, and *Syndinium* sp.^27^, nor in relatives lacking dinomitosis, such as *Amoebophrya*^54,55^ and *Oxyrrhis*^56–58^ (Fig. 5B, Supplementary Figure 3A–C). In sum, putatively NE membrane strands were found in the nuclei of all surveyed dinoflagellates with multiple dinomitotic tunnels, except in *Amphidinium* spp.^25,59^. These are unlikely to be a generalized membrane artifact, as they were only found in mitotic nuclei. Of course, 2D micrographs cannot reveal whether these strands ramify into extensive 3D networks as in *P. kofoidii*, and determining this would require additional tomographic studies or at least serial section TEM analyses of specimens prepared by high pressure freezing. Nevertheless, the presence of strands that interconnect dinomitotic tunnels—all of which are invaginations of the NE—suggests that groups beyond *P. kofoidii* likewise have overlooked membrane complexity. Their appearance during mitosis also begs the question of whether these membrane projections are involved in nuclear division.

Our present description of six tunnels in *P. kofoidii* represents the maximum number of dinomitotic tunnels known, up from five described in *Crypthecodinium cohnii*^28^. Interestingly, across dinoflagellate taxa, the number of dinomitotic tunnels appears to increase in tandem with chromosome counts (Fig. 5B,C). Since the tunnels host membrane-bound kinetochores, adding tunnels could provide additional centromere attachment sites to avoid chromatid overcrowding and mis-sorting during segregation. The nuclear net, by contrast, does not seem to associate with the chromosomes. We found it to be most dense in the equatorial region of the dinokaryon—i.e., the future division plane—where it co-localized with centrin, and could potentially be involved in nuclear division. Yet this would not explain the extensive branches of the net elsewhere in the nucleus. Thus, the nuclear net further contributes to the tangle of unexplained features present in the dinokaryon.

## Conclusion

Our study illustrates a new extreme of membrane complexity in the nuclear envelope, despite it being one of the most conservative eukaryotic features. Our approach also underscores the advantage of using high pressure freezing in conjunction with tomography for visualizing delicate membrane networks. While the NE architecture in dinoflagellates beyond *P. kofoidii* and *C. cohnii* remains to be reconstructed in 3D, our consideration of the 2D ultrastructural literature indicates that NE extensions are widespread among core dinoflagellates, where they bridge neighboring dinomitotic tunnels. These interconnections are surprising, as they would seem to complicate the task of segregating chromosomes along the NE tunnels without them becoming tangled in these strands. Mitosis would seem especially challenging in *P. kofoidii*, where hundreds of chromatids, sliding along six interlinked tunnels, must segregate before each cell division. Future studies should seek a fluorescent marker for the nuclear net, as this could allow live imaging and would address questions about its dynamic behaviors. For instance, when is the nuclear net assembled and does it break down to allow for chromosome segregation? Super-resolution light microscopy could also certify that the nuclear net co-localizes with centrin at the plane of nuclear division, and would help illuminate its interconnections with other elements of the nucleoskeleton. Even in humans, the function of the nucleoskeleton is for the most part elusive^60^, and understanding how this tangle of proteins evolved to maintain our own chromatin—much less that of dinoflagellates—will benefit from such investigations across the tree of life.

## Methods

### Collection

Cells of *Polykrikos kofoidii* were collected off a pier in Vancouver, BC, Canada (49.272704, −123.187827) in July 2015 and off the dock of Friday Harbor Labs, Washington, USA (48.545755,−123.012741) in June 2016 by towing a 20 micron mesh plankton net through surface water. Contents were immediately passed through a 150 micron mesh filter to exclude larger organisms, leaving in a fraction that consisted mostly of predatory dinoflagellates. Within four hours of collection, cells were visually identified under an inverted light microscope and individually isolated by pulled glass micropipettes into dishes of filtered seawater. The isolated cells were colorless binucleate “pseudocolonies” with nematocysts and four transverse flagella (Fig. 1A).

### Transmission electron microscopy (TEM)

Each isolated cell of *Polykrikos kofoidii* was micropipetted onto a flexible Thermonox slide (Fahlenbach, Germany) that had been coated with dried poly-L-lysine to help the specimen adhere. Each cell was fixed in a droplet of a freshly prepared mixture of filtered seawater with 2% glutaraldehyde and 1% OsO_4_ for 30 min. on ice. Most of the mixture was then pipetted away (without drying out the cell) and replaced with a droplet of low melting point agarose that had been preheated to 70 °C (in order to liquify it) then cooled to ~40 °C before being applied to the specimen—at which point the agarose solidified around the cell. This optional step served to further bind the cell to the slide and prevent it from being lost. The slide could then be inverted, suspending the specimen over a well dish, where it was steeped in two rounds of filtered seawater, then dehydrated through a graded series of ethanol (50%, 70%, 85%, 90%, 95%, 100%, 100%), at 10 min each. The slide was then turned face up and the specimen was infiltrated with acetone-resin mixtures (acetone, 2:1, 1:1, 1:2, Epon 812 resin) at 20 min each, then steeped in fresh resin overnight. The resin was then polymerized at 60 °C for at least 24 h. Using a razor, resin was cut away to a 1 mm^3^ block around the cell, which was then carefully removed from the Thermonox slide. This block, containing a single cell, was super glued to a resin stub in the desired orientation for sectioning. Thin (40 nm) sections were produced with a diamond knife, post-stained with uranyl acetate and lead citrate and viewed under a Hitachi H7600 TEM. Four pseudocolonies of *P. kofoidii* were imaged with TEM.

### Focused Ion Beam Scanning Electron Microscopy (FIB-SEM)

Cells of *Polykrikos kofoidii* were individually transferred into a droplet of filtered seawater and frozen immediately to minimize fixation artifacts, using a Leica EM HPM 100 high-pressure freezer (Leica, Wetzlar, Germany). Freeze substitution was used to remove the aqueous content of the cells and replace it with an acetone solution containing 0.5% water, 1% osmium tetroxide and 0.1% uranyl acetate, at −80 °C for 48 h, −20 °C for 6 h, then graded back to 4 °C over 13 h. The prepared samples were washed twice in 100% acetone. Two cells were recovered by micropipette. Each cell was placed on a separate Thermonox coverslip, where it adhered to a patch of poly-L-lysine. Cells were infiltrated with a 1:1 mix of acetone and Embed 812 resin for 2 h, then 100% resin overnight. A second Thermonox coverslip was applied, sandwiching each cell in a thin layer of resin between the coverslips. One cell was accidentally crushed during this process and subsequently discarded. Resin was polymerized at 65 °C for 24 h. Afterwards, the top coverslip was removed with a razor blade to expose the resin face overlying the cell. A single cell of *P. kofoidii* was imaged using an FEI Helios NanoLab 650 dual beam FIB-SEM. The specimen was protected with a 10–20 nm thick gold layer. The SEM beam had a 3.00 kV accelerating voltage and a 30 µs dwell time. The ion beam milled through the cell in 250 nm increments, yielding 946 sections. Images were aligned as a z-stack in Amira 5.5, and structures of interest (chromosomes and the nuclear envelope) were rendered as isosurfaces per manufacturer’s instructions.

### Confocal Microscopy

Cells of *Polykrikos kofoidii* were fixed in 4% paraformaldehyde in filtered seawater for 10 min, then rinsed three times in 0.1 M phosphate buffered saline (PBS) solution before storage in PBS with 0.05% NaN_3_ (sodium azide, as a preservative) at 4 °C. Fixed cells were washed in PBS:NaN_3_ solution with 3 × 15 min exchanges of 0.1 M PBS, followed by permeabilization in PBT (0.1 M PBS + 0.1% Triton X-100) for 30 min at 4 °C. Specimens were triple stained (for tubulin, centrin, and DNA) as follows: Cells were incubated in blocking solution (PBT + 1% bovine serum albumin) at 4 °C for 30 min. Primary staining was then carried out using a mouse anti-tubulin acetylated antibody (Sigma-Aldrich) as well as a rabbit anti-centrin acetylated antibody (against centrins from *Toxoplasma gondii*; Kerafast), each at a concentration of 1:100 in blocking solution. After incubation in the primary antibodies for 12 h at 4 °C, specimens were washed by multiple exchanges of PBT. Secondary staining was then carried out using an anti-mouse Alexa Fluor® 647 antibody (Molecular Probes) and an anti-rabbit Alexa Fluor® 488 antibody, each at a concentration of 1:100 in blocking solution. After incubation in the secondary antibodies for 12 h at 4 °C, specimens were washed by multiple exchanges of PBT. DNA was then labeled by incubating the cells in a 1:100 dilution of Hoechst for 1 h followed by 3 × 15 min exchanges of PBS prior to imaging by confocal laser scanning microscopy. Incubations were always performed in the dark while rocking at 4 °C in glass well plates. Eight pseudocolonies were imaged via confocal. Antibodies are available upon request.

## Supplementary information

Supplementary Figures

## Data Availability

All immunolabelling reagents and original confocal and FIB-SEM images are available, upon request, from the lead author.
