# Supplementary material for: Dinoflagellate nucleus contains an extensive endomembrane network, the nuclear net
Source: Sci Rep. 2019 Jan 29;9:839. doi: 10.1038/s41598-018-37065-w (PMC6351617; doi:10.1038/s41598-018-37065-w)
Supplement: Supplementary file 1 — Supplementary Figures [file 41598_2018_37065_MOESM1_ESM.pdf]

# Dinoflagellate nucleus contains an extensive endomembrane network, the nuclear net.

Gregory S. Gavelis*, Maria Herranz, Kevin C. Wakeman, Christina Ripken, Satoshi Mitarai, Gillian H. Gile, Patrick J. Keeling, & Brian S. Leander

*Corresponding Author

**------------------------------------Supplementary Materials------------------------------------**

**Supplementary Figure 1.**

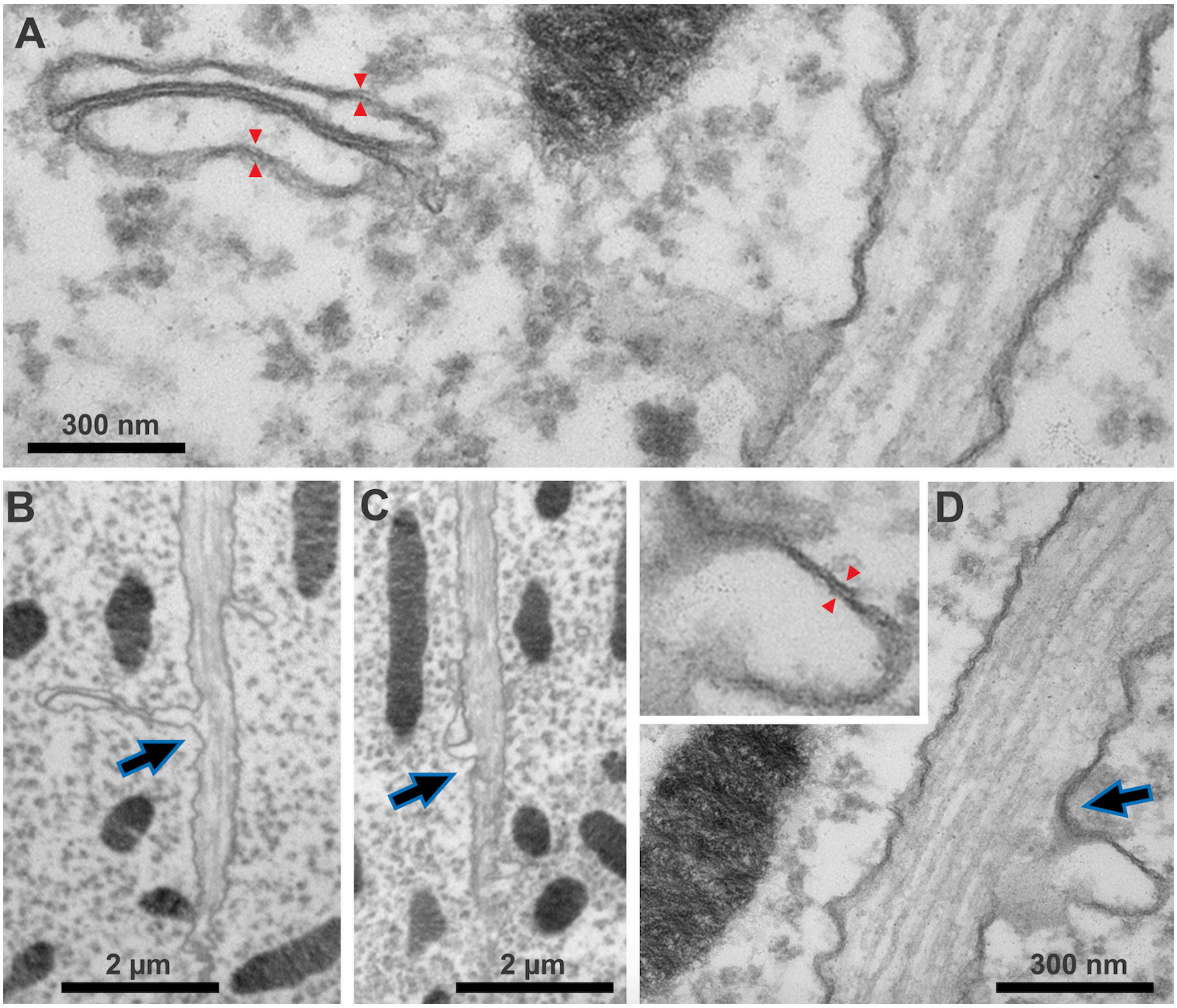


**S1 Legend: The nuclear net is lined by a double membrane sheath.** TEMs of cells prepared by standard fixation, with dinomitotic tunnels in longitudinal section (**A**). Two nodes of the nuclear net are closely appressed, and each is bounded by two membranes (red arrowheads). (**B-D**). Strands of the nuclear net are continuous with the dinomitotic tunnels. The branch point (blue arrow) of each strand is at an approximately right angle to the dinomitotic tunnel. Lumens of the strands and tunnels are likewise continuous, as are the double-membranes (red arrows) that line them.

**Supplementary Figure 2:**

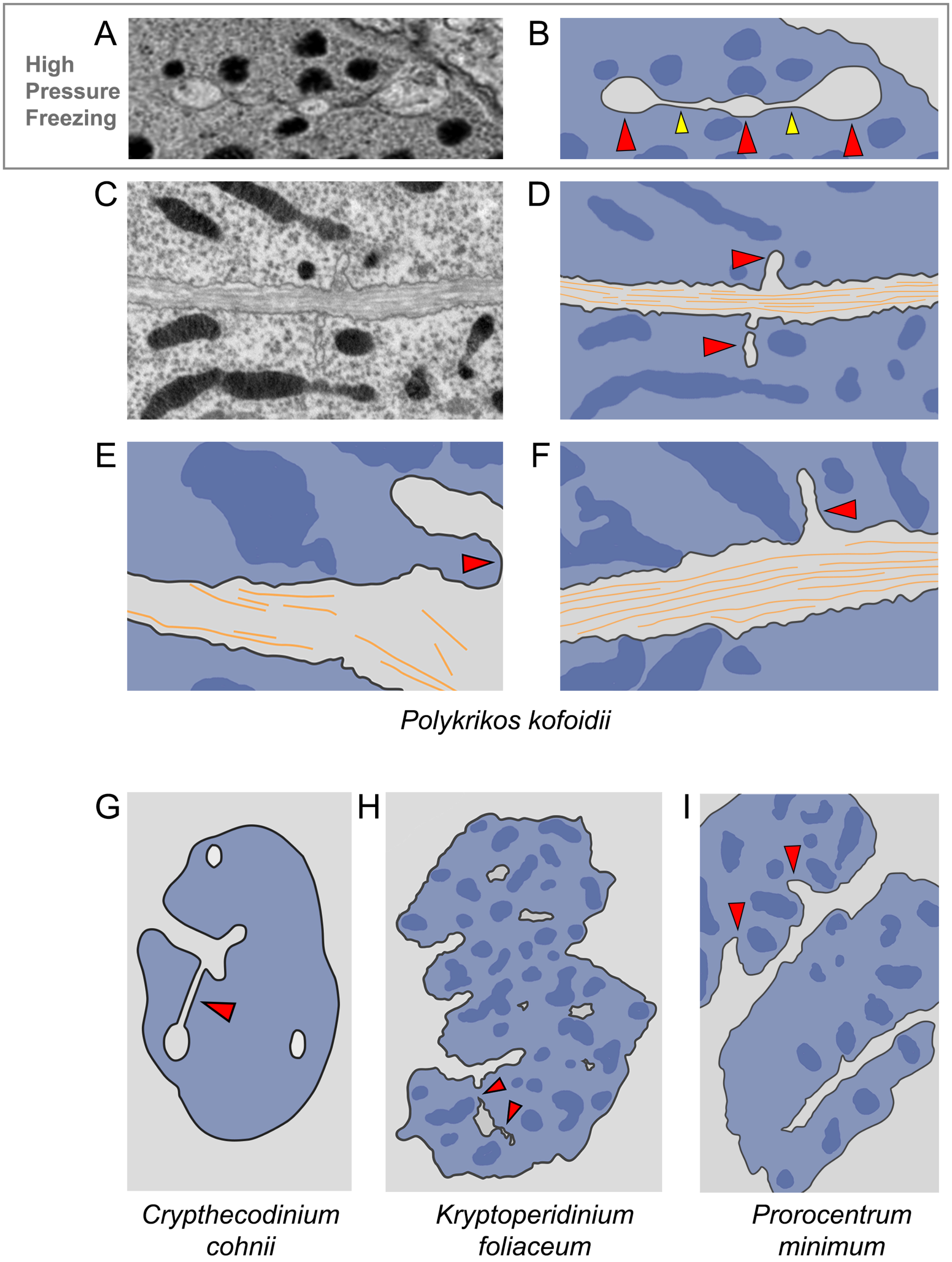

**S2 Legend: Summary of dinoflagellate nuclei that showed evidence of nuclear nets in this study (A-D) and previous (E-I) descriptions.** All images reflect TEMs of cells prepared by standard fixation, except for A-B, in which a cell was prepared by high-pressure freezing and imaged by FIB-SEM (**B**). Swellings (red arrowheads) of the nuclear net are evident in all specimens, but fine strands (yellow arrowheads) connecting the nuclear net were only visible in our high-pressure frozen cell of *Polykrikos kofoidii* (**A, B**). Loss of fine strands in chemically fixed cells from the same population (**C, D**), suggests that high-pressure freezing is necessary to keep the nuclear net intact. **E, F**: traced from Spector & Treimer 1981[44]. **G**: redrawn from a diagram in Kubai and Ris 1969[28]. **H**: traced from Whatley 1993[50]. **I**: traced from Fritz & Triemer 1983[48]. Grey = cytoplasm; blue = nucleoplasm; dark blue = chromosomes; orange = microtubules (source image magnifications for figures B & G-I were too low to resolve microtubules.)

**Supplementary Figure 3:**

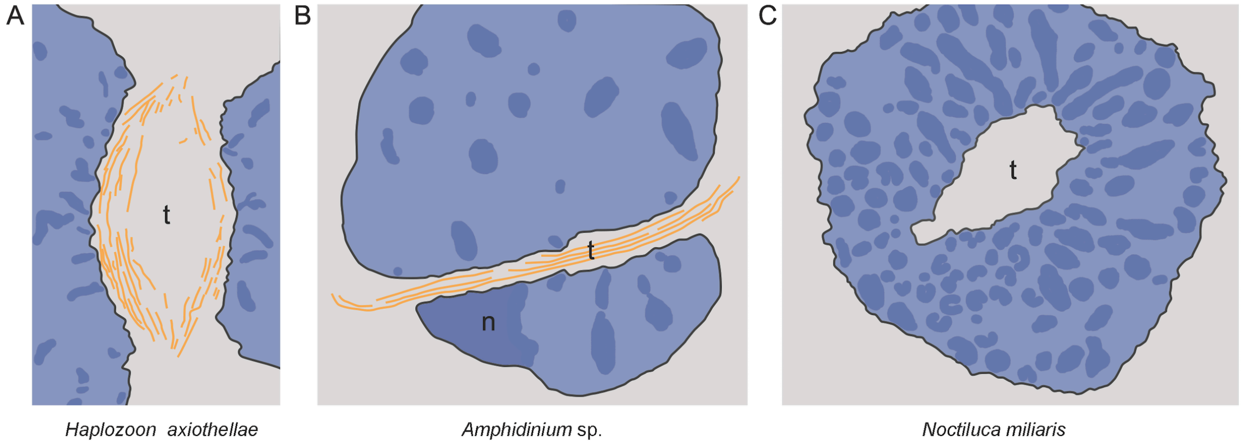


**S3 Legend: Summary of "typical" mitotic dinoflagellate nuclei (i.e., without nuclear nets), from previous TEM literature descriptions.** Nuclei in **A**, **B**, and **C** were traced from TEMs published by Siebert and West 1974[53], Oakley and Dodge 1974[25], and Soyer-Gobillard 1969[51] respectively, from dinokarya that were chemically fixed while undergoing mitosis. t = cytoplasmic tunnels, n = nucleolus. Grey = cytoplasm; blue = nucleoplasm; dark blue = chromosomes; orange = microtubules (source image magnification for Figure C was too low to resolve microtubules).
